# Serum cytokine change profile associated with HBsAg loss during combination therapy with PEG-IFN-α in NAs-suppressed chronic hepatitis B patients

**DOI:** 10.3389/fimmu.2023.1121778

**Published:** 2023-01-23

**Authors:** Wen-Xin Wang, Rui Jia, Xue-Yuan Jin, Xiaoyan Li, Shuang-Nan Zhou, Xiao-Ning Zhang, Chun-Bao Zhou, Fu-Sheng Wang, Junliang Fu

**Affiliations:** ^1^ Senior Department of Infectious Diseases, The Fifth Medical Center of Chinese PLA General Hospital, Peking University 302 Clinical Medical School, National Clinical Research Center for Infectious Diseases, Beijing, China; ^2^ Department of Gastroenterology, The 985th Hospital of Joint Logistic Support Force of Chinese PLA, Taiyuan, China; ^3^ Medical School of Chinese PLA, Beijing, China

**Keywords:** chronic hepatitis B, cytokine, CXCL-10, HBsAg loss, combined treatment, Peg-IFN-α

## Abstract

**Objective:**

The aim of this study was to explore the profile of cytokine changes during the combination therapy with pegylated interferon alpha (PEG-IFN-α) and its relationship with HBsAg loss in nucleos(t)ide analogs (NAs)-suppressed chronic hepatitis B patients.

**Methods:**

Seventy-six patients with chronic hepatitis B with HBsAg less than 1,500 IU/ml and HBV DNA negative after receiving ≥ 1-year NAs therapy were enrolled. Eighteen patients continued to take NAs monotherapy (the NAs group), and 58 patients received combination therapy with NAs and PEG-IFN-α (the Add-on group). The levels of IFNG, IL1B, IL1RN, IL2, IL4, IL6, IL10, IL12A, IL17A, CCL2, CCL3, CCL5, CXCL8, CXCL10, TNF, and CSF2 in peripheral blood during treatment were detected.

**Results:**

At week 48, 0.00% (0/18) in the NAs group and 25.86% (15/58) in the Add-on group achieved HBsAg loss. During 48 weeks of combined treatment, there was a transitory increase in the levels of ALT, IL1RN, IL2, and CCL2. Compared to the NAs group, CXCL8 and CXCL10 in the Add-on group remain higher after rising, yet CCL3 showed a continuously increasing trend. Mild and early increases in IL1B, CCL3, IL17A, IL2, IL4, IL6, and CXCL8 were associated with HBsAg loss or decrease >1 log, while sustained high levels of CCL5 and CXCL10 were associated with poor responses to Add-on therapy at week 48.

**Conclusions:**

The serum cytokine change profile is closely related to the response to the combination therapy with PEG-IFN-α and NAs, and may help to reveal the mechanism of functional cure and discover new immunological predictors and new therapeutic targets.

## Introduction

Approximately 290 million people are chronically infected with the hepatitis B virus (HBV) worldwide (1). More than 650,000 people die each year from end-stage liver disease associated with HBV, including liver failure, cirrhosis, and hepatocellular carcinoma (1–3). Achieving the functional cure, meaning both HBV DNA and hepatitis B surface antigen (HBsAg) undetectable (4–6), can significantly improve the disease progression of chronic hepatitis B (CHB) and is considered the ideal endpoint for antiviral treatment (4–7). Nucleos(t)ide analogs (NAs) and interferon (IFN) are the main first-line agents for CHB, which can significantly inhibit HBV replication. However, it is hard to achieve a functional cure with either drug alone. Although many studies have shown that sequential or combination therapy with the two drugs can significantly improve the probability of functional cure in some specific populations, it is still not satisfactory (8–12). Therefore, it is particularly important to explore the mechanisms behind HBsAg loss and to identify predictive markers to expand the population suitable for treatment.

The development of HBV infection is mainly affected by the host’s immune response. In acute infection, 95% of adult patients showed an adequate immune response and eventually cleared the virus. Otherwise, patients can become chronically infected when the host immune response is inadequate or inappropriate (13, 14). As important immune system components, cytokines may introduce immune dysregulation or tolerance and be associated with progression in CHB (15–18). Inflammatory cytokines, such as IFN-α, CXCL8, CXCL9, and CXCL10, can induce inflammatory immune cell recruitment and promote hepatocyte apoptosis in CHB (15, 19–21). For instance, an elevated serum IFN-α and CXCL8 could promote NK-cell-mediated liver cell injury (20, 21), high serum CXCL9 and CXCL10 levels were reported to correlate with the development of hepatitis flares (20–22), IL-2 and IFN-γ were upregulated with high ALT levels (21). The changes of multiple cytokines can more comprehensively and accurately reflect the immune network in the liver to guide clinical treatment better.

It was also demonstrated that in the case of antiviral therapy, changes in serum cytokines were related to the response to antiviral therapy. Li M. et al. reported that an early rise in IFN-α2 levels during pegylated interferon alpha (PEG-IFN-α) treatment was related to functional cure in hepatitis B e antigen (HBeAg) positive CHB patients (23, 24). In addition, CXCL10 has also shown a predictive role in PEG-IFN-α treatment of chronic hepatitis C (25). Still, the detailed relationship among virological and biochemical markers, and cytokines behind different responses to anti-HBV treatment remains unclear.

In this study, we investigated the dynamic changes of serum cytokines levels during PEG-IFN-α add-on therapy in NAs-suppressed CHB patients with HBsAg levels < 1,500 IU/ml, and explored the relationships between the changes of cytokines, the virological response, and the fluctuation of liver inflammation.

## Materials and methods

### Study population and design

This was an open-label, clinical controlled, observational study. The CHB patients were recruited from the Fifth Medical Center of Chinese PLA General Hospital. Subjects aged 18–65 years who had taken NAs drugs for ≥ 1 year and achieved serum HBsAg levels < 1,500 IU/ml and HBV DNA < 20 IU/ml were eligible. Exclusion criteria for recruited patients were co-infected with other hepatitis viruses or human immunodeficiency virus; liver cirrhosis, liver transplantation, or other liver diseases or severe systemic diseases; and IFN, glucocorticoids, or other immunomodulatory therapy in the six months before enrollment. Enrolled subjects were divided into two groups (the NAs group and the Add-on group) according to their choices after being informed of the benefits and risks of PEG-IFN-α therapy. NAs group continued to receive entecavir (ETV) or tenofovir disoproxil fumarate (TDF). The Add-on group was treated with PEG-IFN-α-2b (180 μg once a week) in addition to ETV or TDF. The primary outcome was HBsAg loss or decline > 1 log after 48 weeks of treatment. Patients were divided into three subgroups according to the responses to 48 weeks of the combination therapy. At week 48, patients who achieved HBsAg loss were defined as complete responders (the CR group); patients who achieved HBsAg decreased > 1 log from baseline but remained positive were classified as partial responders (the PR group); patients who achieved HBsAg decreased < 1 log from baseline and still positive were defined as non-responders (the NR group). The study protocol was approved by the Ethics Committee of the Fifth Medical Center of Chinese PLA General Hospital.

### Clinical and laboratory evaluation

Peripheral blood samples were collected from all enrolled patients during the screening period. Samples were then continuously collected after enrollment every 24 weeks (the NAs group) or every 12 weeks (the Add-on group). Serological and biochemical markers of HBV were routinely tested in the central clinical laboratory. Serum HBV DNA levels were determined by the COBAS AmpliPrep/COBAS TaqMan HBV Test (Roche Molecular Systems, Inc, Branchburg, USA). The lower limit for HBV DNA detection was 20 IU/ml. Serum HBsAg levels were quantified by Elecsys HBsAg II quant II (Roche Diagnostics GmbH, Mannheim, Germany). The lower limit for HBsAg detection was 0.05 IU/ml. COBAS e602 (Roche Diagnostics GmbH, Mannheim, Germany) was used to detect HBsAb, HBeAg, and HBeAb levels. Serum levels of IFNG, IL1B, IL1RN, IL2, IL4, IL6, IL10, IL12A, IL17A, CCL2, CCL3, CCL5, CXCL8, CXCL10, TNF, and CSF2 were determined by flow-cytometer using AIMPLEX kit (Aimplex Biosciences, Inc., Beijing, China) according to the manufacturer’s instructions.

### Statistical analysis

SPSS version 25.0 and R version 4.1.2 were used for statistical analyses. Search Tool for the Retrieval of Interacting Genes (STRING) platforms was utilized for protein-protein interaction network (PPIN) analysis (26). Median (quartiles) were reported for continuous variables. The statistical significance of the difference between the two groups was determined using the Mann–Whitney U test, while among three groups using the Kruskal-Wallis H test. Categorical variables were analyzed using the Chi-squared test. Spearman correlation was adopted to determine the correlation between continuous variables. In the bilateral test, *P* < 0.05 was considered to have a statistical difference.

## Results

### Characteristics of enrolled patients

In this open-label, observational, clinical controlled study, seventy-six patients were enrolled. The characteristics of patients were shown in **Table 1**. At baseline, HBsAg levels were comparable between the NAs and Add-on groups. At week 48, 0.00% (0/18) in the NAs group and 25.86% (15/58) in the Add-on group achieved HBsAg loss, respectively.

**Table 1:** Characteristics of the Enrolled Patients.

Indicators	NAs group (n=18)	Add-on group (n=58)	P
Male/female, n	14/4	49/9	0.763
Age(years), median (quartiles)	38.0(34.0, 47.8)	38.5(32.0, 46.8)	0.859
Baseline HBsAg(log_10_IU/ml), median (quartiles)	2.79(2.28, 3.03)	2.59(1.97, 2.95)	0.328
Baseline HBeAg positive, n (%)	8(47.06)	17(30.91)	0.222
Baseline HBeAb positive, n (%)	7 (41.18)	23 (41.82)	0.963
Baseline ALT(U/L), median (quartiles)	19.5(13.0, 25.0)	22.0(17.8, 27.0)	0.409
Baseline AST(U/L), median (quartiles)	24.0(17.5, 27.3)	21.0(19.0, 24.0)	0.363
HBsAg loss at week 48, n (%)	0 (0)	15(25.86)	0.039

HBeAg/HBeAb data were not available for one patient in the NAs group, for three patients in the Add-on group.

Abbreviations: NAs, nucleos(t)ide analogs; Add-on, nucleos(t)ide analogs combined with pegylated-interferon-alpha; HBsAg, hepatitis B surface antigen; HBeAg, hepatitis B e antigen; HBeAb, hepatitis B e antibody; ALT, alanine aminotransferase; AST, aspartate amino transferase.

After 48 weeks of combination therapy, 15 patients with HBsAg loss were assigned to the CR group, 9 patients with HBsAg decreased > 1 log from baseline but still positive were assigned to the PR group, and 34 patients with HBsAg decreased < 1 log from baseline and remain positive were assigned to the NR group.

### The dynamic change of HBsAg and ALT in the NAs and Add-on groups

**Figure 1:**
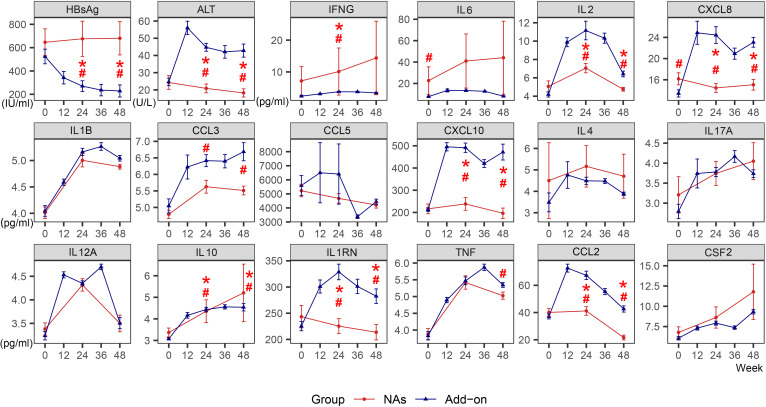
Dynamic changes of HBsAg, ALT and cytokines in the NAs group and Add-on group. The line represents the mean and the bar represents the standard error. #, significant differences in cytokine levels between the two groups, *P*<0.05; *significant differences in the amplitude of cytokine change from baseline between the two groups, *P*<0.05. HBsAg, hepatitis B surface antigen; ALT, alanine aminotransferase; NAs, nucleos(t)ide analogs; Add-on, NAs combined with pegylated interferon alpha therapy.

During 48 weeks of treatment, HBsAg levels declined significantly in the Add-on group, barely dropping in the NAs group. ALT increased more than twofold from baseline at week 12 in the Add-on group and then gradually declined, but the ALT level was still higher than that of the NAs group. ALT in the NAs group was not significantly increased (**Figure 1**).

### The dynamic changes of cytokines induced by PEG-IFN-α are different from those of NAs

The levels of most cytokines, except IFNG and IL6, were lower in the NAs group than in the Add-on group. During 48 weeks of combined treatment, there was a transient spike in the levels of IL1RN, IL2, and CCL2. Compared with the NAs group, CXCL8 and CXCL10 in the Add-on group remain higher after rising, and yet CCL3 showed a continuously increasing trend, and all were significantly higher at week 24 and week 48. (**Figure 1**)

### Mild and early increases in IL1B, CCL3, and IL17A were associated with HBsAg loss or decrease >1 log during combination therapy

**Figure 2:**
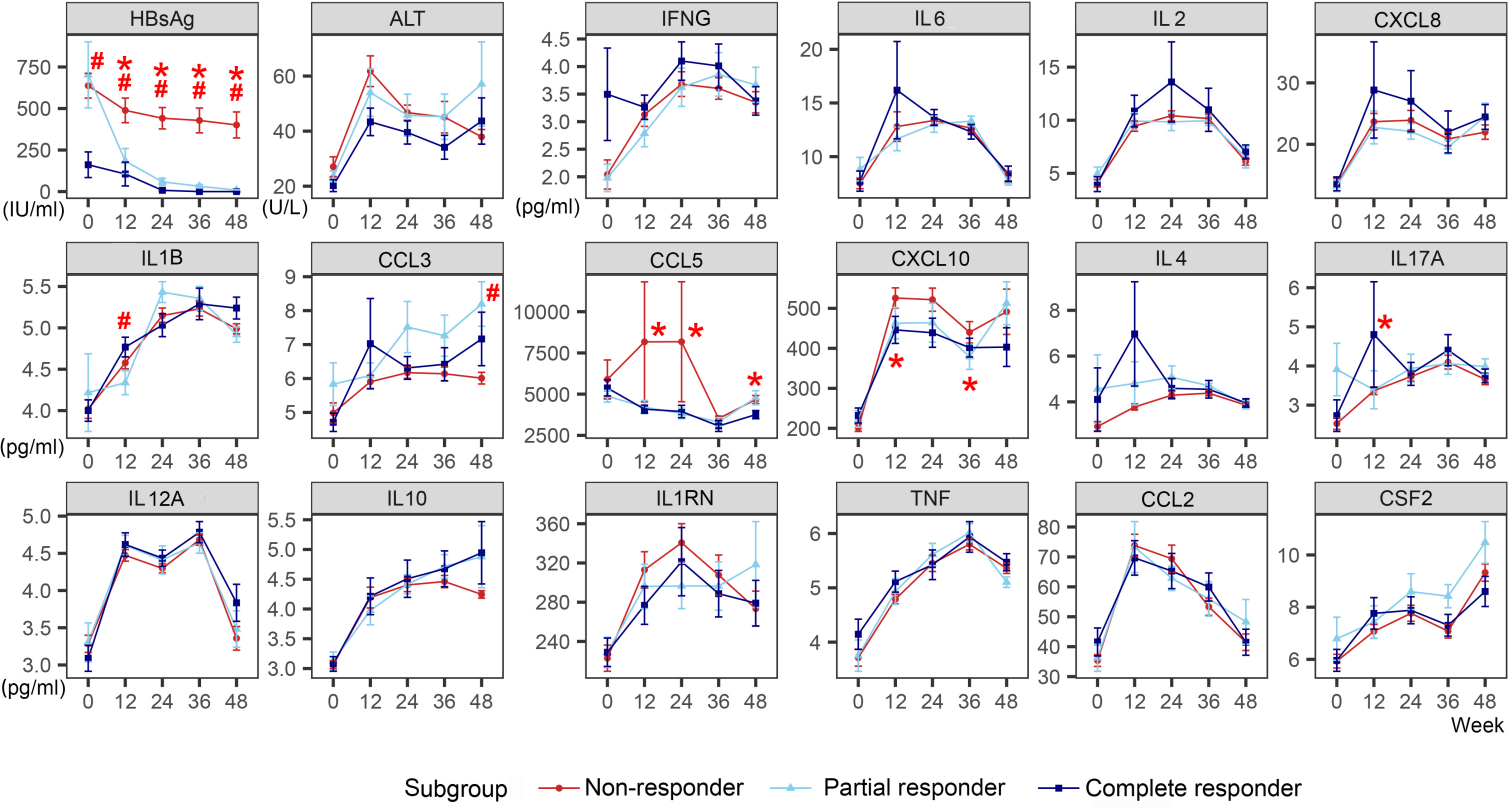
Dynamic changes of HBsAg, ALT, and cytokines of different response subgroups in the Add-on group. The line represents the mean and the bar represents the standard error. #, significant differences in cytokine levels among the three subgroups, *P*<0.05; *significant differences in the amplitude of cytokine change from baseline among the three subgroups, *P*<0.05. HBsAg, hepatitis B surface antigen; ALT, alanine aminotransferase; Add-on, nucleos(t)ide analogs combined with pegylated interferon alpha therapy; complete responder, achieving HBsAg loss at week 48; partial responders: achieving HBsAg decreased by > 1 log from baseline but HBsAg remains positive at week 48; non-responder, achieving HBsAg decreased by < 1 log from baseline and HBsAg remain positive at week 48.

In the Add-on group, ALT peaked at week 12 regardless of the response. Non-responders had the highest ALT peak, with no statistical difference, and then it continued to decline. ALT decreased in the CR and PR groups and then showed a slight upward trend at week 48 (**Figure 2**).

In the CR group, higher baseline levels of IFNG and an early elevation of IL2, IL6, and CXCL8 were observed. The levels of IFNG (week 0), IL2 (week 24), IL6 (week 12), and CXCL8 (week 12) in the CR group were weakly higher than those in the PR and NR group, though with no statistical difference (IFNG [week 0]: H=4.282, *P*=0.118; IL2 [week 24]: H=1.255, *P*=0.534; IL6 [week 12]: H=0.233, *P*=0.890; CXCL8 [week 12]: H=0.822, *P*=0.663). IL17A levels increased significantly at week 12 in the CR group than in the PR and NR groups (H=9.466, *P*=0.009). Typically, at week 12, the CR group showed a transient increase in IL1B and CCL3. IL1B levels increased more in the CR group than in the other patients (H=6.542, *P*=0.038). At week 24, the PR group showed a transient increase in IL1B and CCL3, with no significant difference. However, no visible increase in CCL3 was observed in the NR group. Besides, there was an increase in IL-4 in the CR group at week 12 (H=1.225, *P*=0.542).

In the NR group, the levels of CCL5 and CXCL10 remained high after an obvious increase. At week 12, 24, and 48, the CCL5 level in NR group was significantly higher than that in CR group and PR group (week 12: H=6.361, *P*=0.042; week 24 H=6.409, *P*=0.041; week 48: H=7.584, *P*=0.023). At week 12 and 36, CXCL10 levels increased significantly in the NR group than in the CR and PR group (week 12: H=8.530, *P*=0.014; week 36: H=6.210, *P*=0.045).

Other cytokines, such as IL12A, IL10, TNF, CCL2, and CSF2, showed similar trends regardless of the response to the combination therapy.

### The correlations between the dynamic changes of cytokines, HBsAg, and ALT varied according to the response to combination therapy

**Figure 3:**
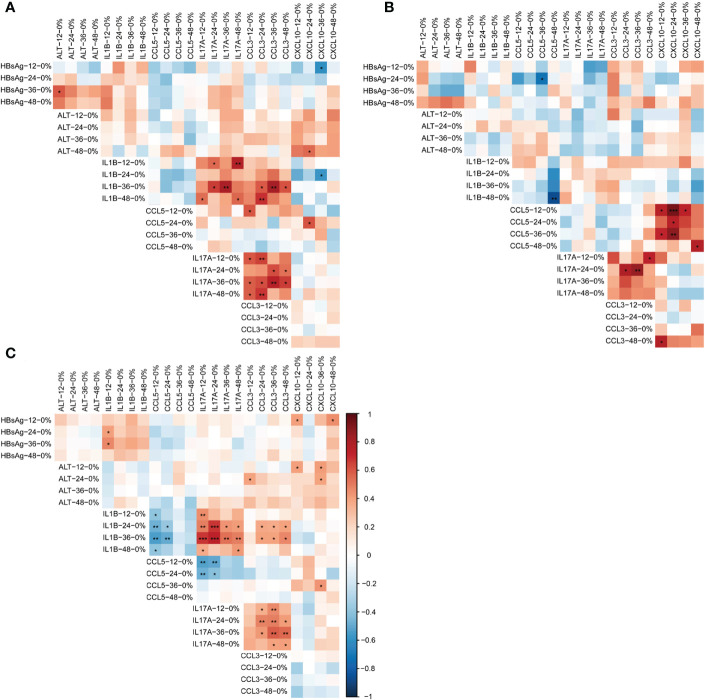
Correlation between dynamic changes of HBsAg, ALT and cytokines in different response to the add-on therapy. **(A)** Correlation between dynamic changes of HBsAg, ALT and cytokines in complete responders **(B)** Correlation between dynamic changes of HBsAg, ALT and cytokines in partial responders **(C)** Correlation between dynamic changes of HBsAg, ALT and cytokines in non-responders. *P<0.05; **P<0.01. HBsAg, hepatitis B surface antigen; ALT, alanine aminotransferase; Add-on, nucleos(t)ide analogs combined with peg-interferon alpha therapy; complete responder, achieving HBsAg loss at week 48; partial responders: achieving HBsAg decreased by > 1 log from baseline but HBsAg remain positive at week 48; non-responder, achieving HBsAg decreased by < 1 log from baseline and HBsAg remain positive at week 48.

We analyzed the correlation between the dynamic changes of cytokines with HBsAg and ALT. A PPIN was constructed among 16 cytokines involved by the STRING database (**Supplementary Figure 1A**). Cytokines with statistically different expression levels in CR, PR, and NR groups were used to reconstruct a PPIN and included in the correlation analysis (**Supplementary Figure 1B**; **Figures 3A–C**).

The presence of all cytokines in one cluster indicated a strong interaction among each other cytokines. Existing databases and text mining showed that most of the cytokines involved in this study had co-expression relationships. There were laboratory-confirmed interactions between IFNG and TNF, CXCL8 and CCL5 and CCL2, IL2 and IL17A, and CXCL10 and CXCL5. There may be protein homology between CCL5 and CCL3 (**Supplementary Figures 1A–B**). The existing database showed that CXCL10 was co-expressed with IL1B, CCL5, and CCL3. Text mining showed that CXCL10 and IL17A were co-expressed. (**Supplementary Figure 1B**)

In the CR subgroup, the increases of IL1B, IL17A, and CCL3 were positively correlated (*P*<0.05). No correlation existed between the increases of other cytokines, HBsAg, and ALT (**Figure 3A**).

In the PR group, the increase of CCL5 was positively correlated with the increase of CXCL10, and the increase of IL17A was also positively correlated with CCL3 (*P*<0.05). There was no correlation between the dynamics of other cytokines, HBsAg and ALT. Of note, the increase in IL1B at week 48 was significantly inversely related to the magnitude of the increase in CCL5 from baseline (r= - 0.867, *P*= 0.002) (**Figure 3B**).

In the NR group, the decline of HBsAg level was correlated positively with the rise of IL1B and CXCL10 (*P*<0.05). The early elevation of ALT was correlated positively with the elevation of CXCL10 (*P*<0.05). The increase of IL1B was correlated negatively with the increase of CCL5 (*P*<0.05) and was correlated positively with the increase of IL17A and CCL3 (*P*<0.05). In addition, the increase of IL17A was correlated negatively with the increase of CCL5 and positively correlated with the increase of CCL3 (*P*<0.05) (**Figure 3C**).

## Discussion

CHB is an immune-related disease; immune cells and cytokines play an essential part in disease progression. Several studies have demonstrated that baseline quantitative HBsAg < 1,500 IU/ml can effectively predict HBsAg loss after a finite course of IFN-based therapy (27, 28). However, HBsAg alone as a predictor could not fully predict the efficacy (15, 29) nor reflect the liver’s immune status (30). Cytokines in serum can indirectly reflect intrahepatic immune response and inflammation (10). Dynamic detection of multiple cytokines can reflect the immune changes more thoroughly to reveal the mechanism of antiviral therapy. However, the association of cytokines with the response to antiviral treatment is yet to be entirely clear. In this study, we found that the slight early increase of Th1 cytokine (IL2), Th2 cytokine (IL4), Th17 cytokine (IL17A and IL6), and proinflammatory cytokines (IL1B, CCL3, and CXCL8) was associated with HBsAg loss or decrease >1 log in the Add-on group, and sustained high levels of proinflammatory chemokines (CCL5 and CXCL10) were associated with poor response during combination therapy of NAs and PEG-IFN-α.

The incidence of liver cirrhosis and hepatocellular carcinoma is significantly reduced after HBsAg loss, which is the treatment goal of CHB (4, 31, 32). An expert consensus recommends a 1log drop in HBsAg in early treatment as an indication for continued PEG-IFN-α therapy (27). Multiple studies have confirmed that a 1log drop in HBsAg predicts functional cure (33–37). Therefore, we defined good response and partial response as HBsAg loss and decreasing > 1 log, respectively.

In CHB, Th1/Th2 and Th17/Treg were in immune imbalance (30, 38, 39), and antiviral treatment partially restored immune cell function (30). IL2 (Th1 cytokine) activated CD8+T cells and NK cells and promoted their proliferation and cytosolic activity (40–42). NK cells can indirectly regulate T cells by releasing or consuming cytokines (43, 44). Peg-IFN-α increased IL2 activity *in vitro (* 45) and enhanced the inhibition of NK cells on regulatory T cell proliferation and differentiation through IFNG (46). IL4 (Th2 cytokine), produced by NKT cells, inhibits HBV RNA and HBsAg production (47). Activated Th17 cells primarily secrete cytokines such as IL17A, IL6, and TNF and also recruit macrophages, neutrophils, and lymphocytes to induce local inflammation (48–51). IL6 (Th17 cytokine) exerts direct antiviral effects by activating the NF-κB pathway in infected hepatocytes (52, 53). A cross-sectional study found that high levels of IL17A were associated with spontaneous HBsAg loss (48), which is consistent with our study. IL-17 could effectively inhibit HBV replication in a noncytopathic manner (54). IL4 has been confirmed to inhibit the secretion of IL6 and TNF *in vivo* and *in vitro (* 55). In this study, IL2, IL4, IL6, and IL17A (Th17 cytokine) in the Add-on group showed the same change trend, increasing at the early stage and decreasing at the later stage, especially in patients with good response, suggesting that the dynamic balance between Th1/Th2/Th17 immunity may play a key role in functional cure under combination therapy.

Especially, the timing of CCL3 and IL1B elevation may be associated with the response of antiviral therapy. During the combination therapy, patients with an increase in IL1B and CCL3 at week 24 did not respond as well as those with an increase at week 12, but the non-responders had almost no significant increase in CCL3. IL1B is a pro-inflammatory and antiviral cytokine (52, 56), produced mainly by inflammatory macrophages in the liver (52). HBV modulates liver macrophage function to impair the production of IL1B to maintain the infection status (52). It is speculated that the late or insignificant increase in IL1B may be a manifestation of an inhibitory immune microenvironment, which is not conducive to the functional cure of CHB.

Compared with the CR and PR groups, CCL5 and CXCL10 were maintained at high levels after the early increase in the NR group during combined treatment. CXCL10 is a potent chemoattractant of activated T cells, leading to immune activation (48). Sonneveld MJ et al. observed that higher levels of CXCL10 before PEG-IFN therapy may be beneficial for obtaining HBeAg loss (57). However, Wong, G.L. et al. reported that lower levels of CXCL10 can predict HBsAg loss (58), which was consistent with our studies. In fact, 41 HBeAg-positive and 45 HBeAg-negative patients were enrolled and received 48 weeks of PEG-IFN-α plus adefovir. And S B Willemse et al. figured that higher baseline CXCL10 levels appeared to be associated with favorable responses in HBeAg-positive patients but not in HBeAg-negative patients (13). Therefore, the relationship between cytokines and the response may be influenced by HBV replication levels. However, in this study, CXCL10 did not show significant changes during NAs treatment, which may be a manifestation of the lack of an effective immune response.

We noted that the level of IFNG was higher in patients with good responses than in patients with partial responses and poor responses under combination therapy. But patients treated with NAs had higher levels of IFNG and worse responses. This phenomenon may be related to the duration of high levels of IFNG. It has been reported that the presence of long-term IFNG is associated with chronic inflammation and contributes to the occurrence of tumors (59). In the Add-on group, the IFNG level increased and then decreased. It is suggested that long-term high levels of pro-inflammatory cytokines are not conducive to the functional cure, and the dynamic balance between pro-inflammatory and anti-inflammatory cytokines plays a vital role in treating CHB.

CHB is characterized by immunosuppression, decreased anti-inflammatory cytokines expression, and increased anti-inflammatory cytokines expression. The ability of NK cells to secrete cytokines and the HBV-specific immune response was partially restored after the immune tolerance was broken (60). The PEG-IFN-α treatment restored immunity and suppressed the production of immunosuppressive cytokines (61). Li, M. H. et al. (61) enrolled patients with CHB and treated them with NAs and PEG-IFN-α, respectively. It was found that the level of IL10 in both groups increased with time, and the increase was more evident in the NAs group, which was consistent with our study. In our study, the level of IL10 increased in both the NAs and Add-on treatment, especially during NA treatment.

The different correlations of cytokines among patients with different responses to PEG-IFN-α treatment are due to different profiles of cytokine change. Ning Qin et al. found that PEG-IFN-α enhanced the inhibition of NK cells on Treg cells, and this inhibition was associated with a significant decline in HBsAg (46). Nishio, A. et al. found that patients with early NK cell activation after PEG-IFN-α treatment had greater HBsAg decline (62). Our study suggests that PEG-IFN-α treatment induces different immune microenvironment changes, which may be the mechanism of different outcomes. But the underlying mechanism needs further exploration. There was a significant inverse correlation between IL1B and CCL5 in nonresponsive patients. IL1B is a proinflammatory cytokine associated with innate immunity. CCL5 is mainly produced by CD8^+^T cells and can chemotaxis and activate T lymphocytes, dendritic cells, and natural killer cells (63). The negative correlation between the two cytokines in non-responders may indicate that the coordinated interaction between innate and specific immunity contributes to viral clearance.

In summary, our study found that the pattern of serum cytokine dynamics correlates with the response to the sequential combination therapy of PEG-IFN-α and NAs. A mild and harmonious interaction of Th1/Th2/Th17 cytokines is associated with a favorable virological response, and sustained high levels of certain inflammatory cytokines are not conducive to HBsAg clearance. There were some deficiencies in this study. First, we did not detect the variation in the number and function of cytokine-related immune cells. Second, cytokines and HBsAg in liver tissue were not detected. However, these preliminary results provide clues for further revealing the immune mechanism of antiviral therapy and developing new therapeutic targets.

## Data availability statement

The raw data supporting the conclusions of this article will be made available by the authors, without undue reservation.

## Ethics statement

The study protocol was approved by the Ethics Committee of the Fifth Medical Center of Chinese PLA General Hospital. The patients/participants provided their written informed consent to participate in this study.

## Author contributions

JF contributed to conception and design of the study. X-YJ, XL, S-NZ, X-NZ, C-BZ participated in data collection. W-XW performed the statistical analysis. W-XW, RJ wrote the first draft of the manuscript. F-SW and JF revised the manuscript. All authors contributed to the article and approved the submitted version.
