# Supplementary figures and images for: Serum cytokine change profile associated with HBsAg loss during combination therapy with PEG-IFN-α in NAs-suppressed chronic hepatitis B patients
Source: Front Immunol. 2023 Jan 23;14:1121778. doi: 10.3389/fimmu.2023.1121778 (PMC9899895; doi:10.3389/fimmu.2023.1121778)

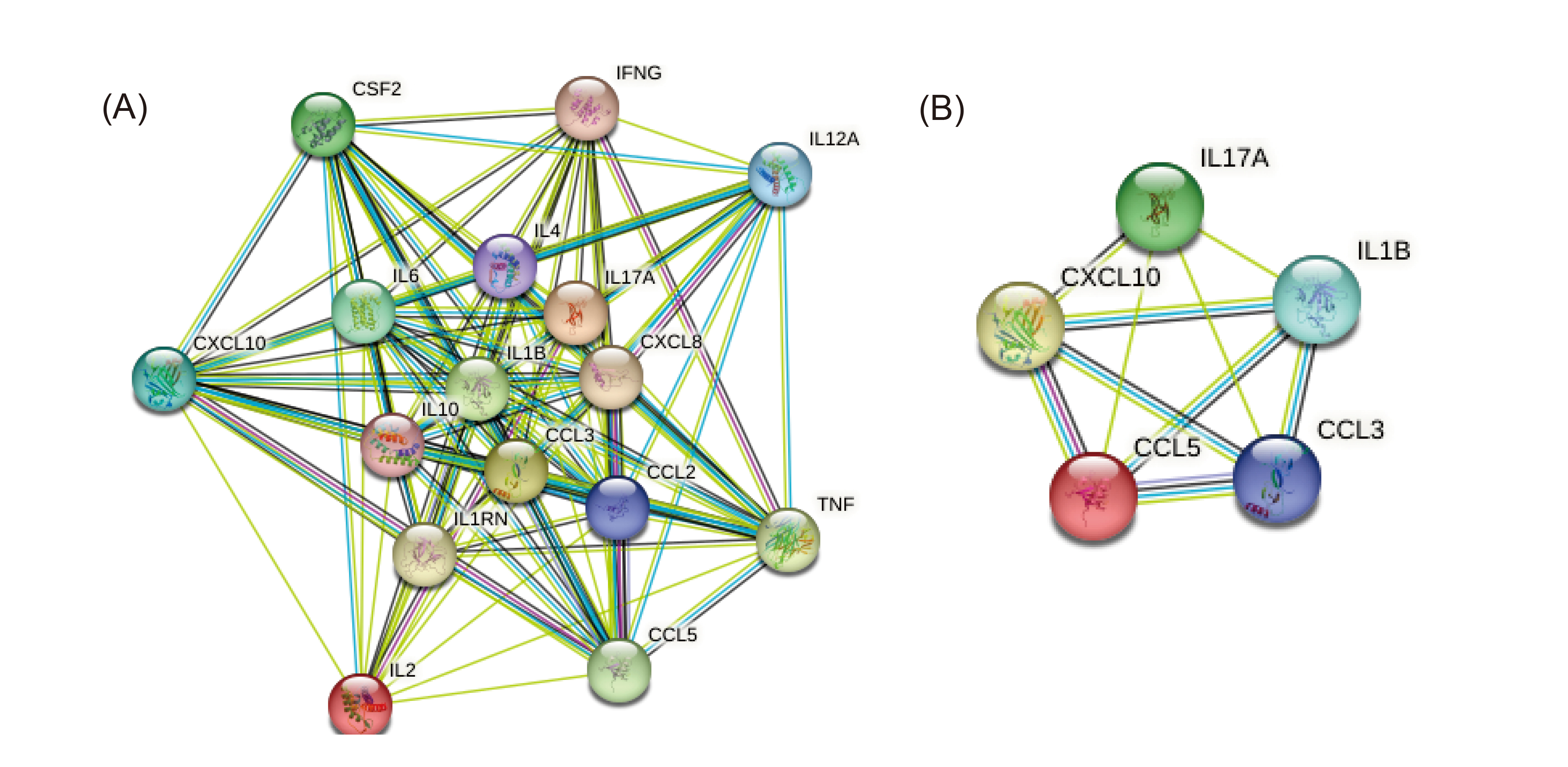

Supplement: Supplementary file 1 [file Image_1.jpeg]
